# Kvik: three-tier data exploration tools for flexible analysis of genomic data in epidemiological studies

**DOI:** 10.12688/f1000research.6238.2

**Published:** 2015-06-16

**Authors:** Bjørn Fjukstad, Karina Standahl Olsen, Mie Jareid, Eiliv Lund, Lars Ailo Bongo

**Affiliations:** 1 Department of Computer Science, UiT - The Arctic University of Norway, Tromsø, 9037, Norway; 2 Department of Community Medicine, UiT - The Arctic University of Norway, Tromsø, 9037, Norway

**Keywords:** Functional genomics, Epidemiological studies, Data exploration, On-demand data analysis, Open-source software, Kvik

## Abstract

Kvik is an open-source framework that we developed for explorative analysis of functional genomics data from large epidemiological studies. Creating such studies requires a significant amount of time and resources. It is therefore usual to reuse the data from one study for several research projects. Often each project requires implementing new analysis code, integration with specific knowledge bases, and specific visualizations. Although existing data exploration tools are available for single study data exploration, no tool provides all the required functionality for multistudy data exploration. We have therefore used the Kvik framework to develop Kvik Pathways, an application for exploring gene expression data in the context of biological pathways. We have used Kvik Pathways to explore data from both a cross-sectional study design and a case-control study within the Norwegian Women and Cancer (NOWAC) cohort. Kvik Pathways follows the three-tier architecture in web applications using a powerful back-end for statistical analyses and retrieval of metadata.In this note, we describe how we used the Kvik framework to develop the Kvik Pathways application. Kvik Pathways was used by our team of epidemiologists toexplore gene expression data from healthy women with high and low plasma ratios of essential fatty acids.

## Introduction

Visual explorative analysis is essential for understanding biological functions in large-scale omics’ datasets. However, enabling the inclusion of omics’ data in large epidemiological studies requires collecting samples from thousands of people at different biological levels over a long period of time. It is therefore usual to reuse the data for different research questions and projects. Although an existing tool may be useful for one project, no tool provides the required functionality for several different projects.

We have designed and implemented Kvik, a framework that makes it easy to develop new applications to explore different research questions and data. The initial version Kvik ^1^ contained a prototype system for exploring biological pathways and gene expression data. From this prototype we built the Kvik Framework, which provides developers a simple interface to powerful systems for statistical analyses and meta-databases, and Kvik Pathways: a publicly available data exploration application. From our experience in developing a framework for building data exploration applications, we identified four requirements such applications should satisfy:

**Interactive** The applications should provide interactive exploration of datasets through visualizations and integration with relevant information. To understand the large quantities of heterogeneous data in epidemiological studies, researchers need interactive visualizations that provide different views and presentations of the data. Also, to understand the results it is important to have instant access to existing knowledge from online databases.
**Familiar** They should use familiar visual representations to present information to researchers. For more efficient data exploration it is effective to use representations that researchers are familiar with both from the literature and from other applications.
**Simple to use** Researchers should not need to install software to explore their data through the applications. The applications should protect the researcher from the burden of installing and keeping an application up to date.
**Lightweight** Data presentation and computation should be separated to make it possible for researchers to explore data without having to have the computational power to run the analyses. With the growing rate data is produced at, we cannot expect that researchers have the resources to store and analyze data on their own computers.

There are several tools for exploring biological data in the context of pathways, such as VisANT (available online at visant.bu.edu) by 2, VANTED (available online at vanted.ipk-gatersleben.de) ^3^, enRoute by 4 or Entourage by 5 (both available online at caleydo.org). However, these tools do not provide the adaptability needed for exploration of multi-study datasets. Many existing tools place the visualization, data analysis and storage on the user’s computer, making it necessary to have a powerful computer. In addition, the tools are often standalone applications that require users to install and update the applications. Kvik Pathways satisfies the above requirements as follows:

**Interactive** Kvik Pathways provides interactive pathway visualizations and information from the popular Kyoto encyclopedia of genes and genomes (KEGG) ^6^ database (available online at kegg.jp).
**Simple to use** Kvik Pathways uses HTML5 and modern JavaScript libraries to provide an interactive application that runs in any modern web browser.
**Familiar** Kvik Pathways uses the familiar pathway representations from KEGG and graphical user interfaces found in modern web applications.
**Lightweight** Kvik Pathways uses a powerful back-end provided by the Kvik framework to perform statistical analyses.

Both Kvik and Kvik Pathways are open-sourced at github.com/fjukstad/kvik. We provide an online version of Kvik Pathways at kvik.cs.uit.no and to run Kvik Pathways in a local Docker instance or on a cloud service such as Amazon Web Services ( aws.amazon.com) or Google Compute Engine ( cloud.google.com/compute), we provide a Docker image at registry.hub.docker.com/u/fjukstad/kvik.

In this note we describe how we used Kvik to implement Kvik Pathways, a tool for exploring gene expression in the context of biological pathways. In Kvik Pathways researchers can explore gene expression data from 7 combined with information from online knowledge bases. We provide the following contributions:

- Kvik Pathways, a publicly available web application for exploring gene expression data in the context of biological pathways without any additional applications than a web browser.
- A requirement analysis for interactive exploration tools for epidemiological studies.
- A detailed description of how we have used Kvik Pathways to explore gene expression data from healthy women with high and low plasma ratios of essential fatty acids.

## Methods

**Figure 1.**
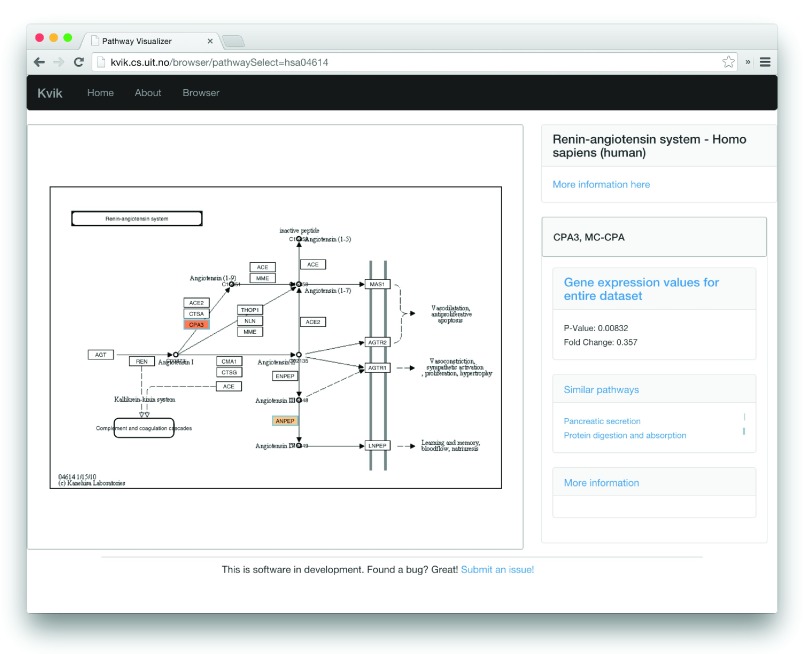
Screenshot of the renin-angiotensin pathway (KEGG pathway id hsa04614) in Kvik Pathways. The user has selected the gene CPA3, which brings up the panel on the right. From here researchers can browse pathways that the gene is a member of, and read relevant information about the gene from KEGG.

Kvik Pathways allows users to interactively explore a molecular dataset, such as gene expression, through a web application. It provides pathway visualizations and detailed information about genes and pathways from the KEGG databases ( Figure 1). Through pathway visualizations and integration with the KEGG databases, epidemiologists can perform targeted exploration of pathways and genes to get an overview of the biological functions that are involved with gene expression from the underlying dataset. Kvik Pathways gathers information about related pathways and retrieves relevant information about genes, making it unnecessary for researchers to spend valuable time looking up this information manually. For example, navigating a set of pathways and browsing information about genes in these, requires the researcher to manually query KEGG for each specific gene. Kvik Pathways retrieves information about genes without the researcher having to leave the pathway visualization to retrieve relevant information.

The Kvik framework provides a flexible statistics back-end where researchers can specify the analyses they want to run to generate data for later visualization. For example, in Kvik Pathways we retrieve fold change for single genes every time a pathway is viewed in the application. These analyses are run ad hoc on the back-end servers and generates output that is displayed in the pathways in the client’s web browser. The data analyses are implemented in a simple R script and can make use of all available libraries in R, such as Bioconductor ( bioconductor.org).

Researchers modify this R script to, for example, select a normalization method, or to tune the false discovery rate (FDR) used to adjust the *p*-values that Kvik Pathways uses to highlight significantly differentially expressed genes. Since Kvik Pathways is implemented as a web application and the analyses are run ad hoc, when the analyses change, researchers get an updated application by simply refreshing the Kvik Pathways webpage.

### Implementation

We implemented interactive visualizations using the Cytoscape.js ( js.cytoscape.org) library to generate the interactive pathway visualizations, and D3 ( d3js.org) for Document Object Model (DOM) manipulation such as generating bar charts with HTML < *svg*> elements. We integrate these with the popular Bootstrap front-end framework ( getbootstrap.com) to provide a familiar and aesthetically pleasing user interface.

**Figure 2.**
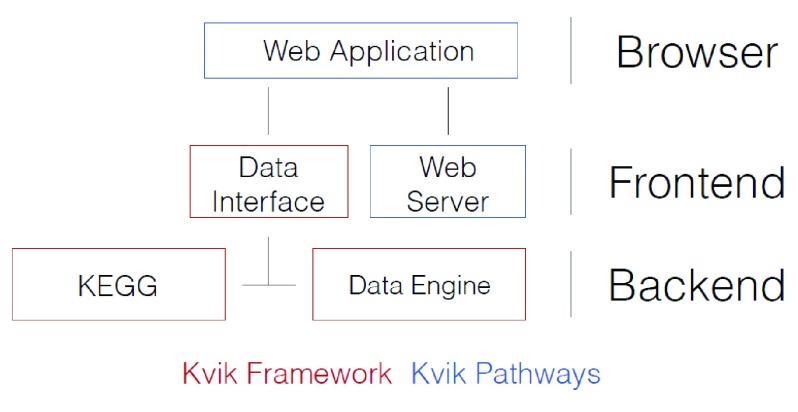
The three-tiered architecture of Kvik Pathways.

Kvik Pathways has a three-tiered architecture of independent layers ( Figure 2). The browser layer consists of the web application for exploring gene expression data and biological pathways. A front-end layer provides static content such as HTML pages and stylesheets, as well as an interface to the data sources with dynamic content such as gene expression data or pathway maps to the web application. The back-end layer contains information about pathways and genes, as well as computational and storage resources to process genomic data such as the NOWAC data repository. The Kvik framework provides the components in the back-end layer.

In our setup the Data Engine in the back-end layer provides an interface to the NOWAC data repository stored on a secure server on our local supercomputer. In Kvik Pathways all gene expression data is stored on the computer that runs the Data Engine. The Data Engine runs an R session accessible over remote procedure calls (RPCs) from the front-end layer using RPy2 ( rpy.sourceforge.net) to interface with R. To access data and run analyses the Data Interface exposes a HTTP API to the browser layer ( Table 1 provides the interfaces).

**Table 1.** The REST interface to the Data Engine. All URLs are relative to the hostname where the Data Engine server runs. On our public installation the Data Engine runs on kvik.cs.uit.no:8888. For example, use kvik.cs.uit.no:8888/genes/ to retrieve all available genes in our dataset. By using a HTTP API we can build different data exploration applications in virtually any programming language.

URL	Description
*/fc/[genes...]*	Calculate and retrieve fold-change for the specified genes
*/pvalues/[genes...]*	Calculate and retrieve *p*-values for the specified genes
*/exprs/[genes...]*	Get the raw gene expression values from the dataset
*/genes*	Get a list of all genes in the dataset

To create pathway visualizations the Kvik back-end retrieves and parses the KEGG Markup Language (KGML) representation and pathway image from KEGG databases through its REST API ( rest.kegg.jp). This KGML representation of a pathway is an XML file that contains a list of nodes (genes, proteins or compounds) and edges (reactions or relations). Kvik parses this file and generates a JSON representation that Kvik Pathway uses to create pathway visualizations. Kvik Pathways Cytoscape.js to create a pathway visualization from the list of nodes and edges and overlay the nodes on the pathway image. To reduce latency when using the KEGG REST API, we cache every response on our servers. We use the average fold change between the groups (women with high or low plasma ratios of essential fatty acids) in the dataset to color the genes within the pathway maps. To highlight *p*-values, the pathway visualization shows an additional colored frame around genes. We visualize fold change values for individual samples as a bar chart in a side panel. This bar chart gives researchers a global view of the fold change in the entire dataset.

### Operation

Kvik Pathways runs in all modern web browsers and does not require any third-party software.

## Use case

We used Kvik Pathways to repeat the analyses in a previous published project ( 7, doi: 10.1371/journal.pone.0067270) that compared gene expression in blood from healthy women with high and low plasma ratios of essential fatty acids. Gene expression differences between groups were assessed using *t*-tests ( *p*-values adjusted with the Benjamini-Hochberg method). There were 184 differentially expressed genes significant on the 5% level. When exploring this gene list originally, functional information was retrieved from GeneCards and other repositories, and the list was analyzed for overlap with known pathways using MSigDB (available online at broadinstitute.org/gsea/msigdb). The researchers had to manually maintain overview of single genes, gene networks or pathways, and gather functional information gene by gene while assessing differences in gene expression levels. With this approach, researchers are limited by manual capacity, and the results may be prone to researcher bias. Kvik Pathways eliminates this researcher bias and does not limit the information retrieval to a researcher’s manual capacity.

Initially, Kvik Pathways was implemented to explore gene expression data from a not yet published dataset. To use Kvik Pathways to explore the data from the analyses in 7, we only needed to make small modifications to the analysis R script used by the Data Engine. (The modified R script is found at github.com/fjukstad/kvik/blob/master/dataengine/data-engine.r). Instead of loading the unpublished dataset, we could load the dataset from 7 and use the four functions that are accessible over RPC ( Table 1 shows the HTTP API which uses the underlying RPCs). Currently this script is less than 30 lines, consisting of four functions to retrieve data and a simple initialization step that reads the dataset. Researchers only have to modify these four functions to enable exploration of new datasets. As of the current implementation of Kvik Pathways researchers have to modify the analysis script outside the application.

As an example of practical use of Kvik Pathways, we chose one of the significant pathways from the overlap analysis, the renin-angiotensin pathway (Supplementary table S5 in 7). The pathway contains 17 genes, and in the pathway map we could instantly identify the two genes that drive this result. The color of the gene nodes in the pathway map indicates the fold change, and the statistical significance level is indicated by the color of the node’s frame. We use this image of a biological process to see how these two genes (and their expression levels) are related to other genes in that pathway, giving a biologically more meaningful context as compared to merely seeing the two genes on a list.

## Summary

Kvik Pathways is an open-source system for explorative analyses of functional genomics data from epidemiological studies. It uses R to perform on-demand data analyses providing a flexible back-end that can expand to new analyses and research projects. It uses modern visualization libraries and a powerful back-end for on-demand statistical analyses. Epidemiologists are using Kvik Pathways to analyze gene expression data. Kvik Pathways is open-sourced at github.com/fjukstad/kvik and is available as a Docker image at registry.hub.docker.com/u/fjukstad/kvik.

## Data availability

The data referenced by this article are under copyright with the following copyright statement: Copyright: © 2015 Fjukstad B et al.

Data associated with the article are available under the terms of the Creative Commons Zero "No rights reserved" data waiver (CC0 1.0 Public domain dedication). 

Data used in the use case is available in the Gene Expression Omnibus ( ncbi.nlm.nih.gov/geo), under accession number GSE15289.

## Software availability

### Latest source code

https://github.com/fjukstad/kvik

### Source code as at the time of publication

https://github.com/F1000Research/kvik/releases/tag/1.0

### Archived source code as at the time of publication

http://dx.doi.org/10.5281/zenodo.16375

### Software license

The MIT license.
