# Supplementary material for: Kvik: three-tier data exploration tools for flexible analysis of genomic data in epidemiological studies
Source: F1000Res. 2015 Jun 16;4:81. Originally published 2015 Mar 30. [Version 2] doi: 10.12688/f1000research.6238.2 (PMC4574765; doi:10.12688/f1000research.6238.2)
Supplement: Peer review history

# Peer review: Referee response for version 2

**DOI:** 10.5256/f1000research.7113.r10131

**Published:** 2015-08-26

**Authors:** Lilit Nersisyan

**Affiliations:** 1 Group of Bioinformatics, Institute of Molecular Biology, National Academy of Sciences of Armenia, Yerevan, Armenia

In their paper, Fjukstad *et al., *have described Kvik, a web-based tool intended for KEGG pathways based analysis of gene expression data, and easy sharing and delivery of analysis results to a wide audience via web-based visualization.

***General feedback:***

The authors have tried to separate the knowledge base, from heavy statistical calculations, and those from actual visualization of the results. This would make the software both flexible for modifications, adjustable for growing data, and at the same time, could allow the end user to see the updated version of calculations by simply updating their browser.

While this idea is of great value, the implementation seems not to fully correspond to the intent, and the manuscript fails to deliver the proper functionality, the applications, and the target audience of the software.

I had to refer to the authors’ previous publication, "Kvik: Interactive exploration of genomic data from the NOWAC postgenome biobank" (conference paper), to understand the actual intent of the framework and the Kvik pathways. To my understanding, the current paper adds little value to this previous publication, even with the use case described.

The authors could stress on the actual functionality of Kvik, describe more elaborate use cases, and compare it to existing approaches. The advantages of the tool are not clear: those are mentioned in the introduction and the summary, but do not really correspond to the actual implementation state, and are not addressed in the text body.

***Title*** *:*

The title refers to the *general* idea of three-tier data exploration tools and their value for *general* use in epidemiological studies. However, the paper describes only Kvik pathways developed with their framework, and only a simple mapping of gene expression data onto KEGG pathways.

***Abstract:***

The abstract concentrates on the problem of multiple usages of the same data in different studies, and mentions that Kvik somehow solves this problem. There is no at least understandable message in the text describing how exactly Kvik is a solution.

***Article content:***

The content was hard to comprehend. I’d like to mention the following major points that might be relevant:

 

1. The introduction section is redundant with double notice of the features a good framework should satisfy and which features Kvik itself possesses. Probably the authors could concentrate more on what features they intended to incorporate in Kvik, and why those features are important, in the same paragraph, and describe how exactly these features are satisfied further in the implementation.
2. The introduction itself messes up the target audience: in the beginning it mentions that Kvik is to provide a simple framework for developers to apply advanced statistics on the data, but later on mentions only that the ‘easy to use’ feature of Kvik is for the end-users to see the visualization results.
3. As continuation of the previous comment, the paper fails to describe how exactly a user can modify the statistical analysis algorithms in the R scripts (“outside the application” is not sufficient). This seems to be one of the main features of Kvik, and it is mentioned in the introduction and mostly in the summary, however no further explanations or examples are provided in the body.
4. It is not true that having a browser is everything that is needed for using Kvik. It’s true for exploration of Kvik pathways only, but not for applying custom statistical analysis. Additionally, the users need to be familiar with how to use Docker images, and this again poses the question of who is the target audience.
5. It is confusing to load pathways at http://kvik.cs.uit.no/, and see FC values without knowing what these FC values actually mean. The user does not need to refer to the paper to see which values of which dataset are compared with each other to derive those FC values, and the browser only gives the information that the data is from the NOWAC biobank. Again, the paper fails to fully describe how the R script should be modified to include their own dataset and how these modifications will appear on the browser.
6. It is too slow. With 13 Mbps internet speed, it requires more than 30 seconds to load the list of pathways, and nearly 30 seconds to load a single pathway.
7. In the use case, the comparison of Kvik with the previously published approach is not appropriate. The authors do not clearly distinguish between gene set enrichment and pathway overrepresentation (overlap) analysis. The fact that the researchers should manually lookup gene functions is not solved by Kvik, since the output of Kvik is not the same as the output of those researchers: it simply provides mapping of expression data onto pathways. Moreover, the notion of “restricted to researcher’s manual capacity and the results may be prone to researcher bias” is not addressed by Kvik. Even though the genes appear in the pathway automatically, the researcher still has to scroll through the pathways and subjectively decide what the output means in biological sense. Thus, the output is limited and the bias is still there.
8. There is no comparison with existing tools, except for the sentence *“However, these tools do not provide the adaptability needed for exploration of multi-study datasets” *in the introduction, which is not clear.  

***Summary***

The summary is a good overview of the authors’ initial intent and idea, which is great. However, the summary does not really correspond to the body of the text.

***Minor points***

1. The browser is unresponsive when in the middle of loading one wants to push the back button.
2. Too little data. The majority of genes in the pathways do not have associated FC values. This makes the visualization results not clear. The authors may either mention this in the paper, or provide a richer dataset.
3. It is not clear what is meant by:
   
  1.  “ *required functionality for multistudy data exploration*” in the abstract.
  2. “ *from thousands of people at different biological levels*” in the introduction.
  3. *“A requirement analysis for interactive exploration tools*” in the introduction.
4. The authors should make clear what fold change values are used exactly, when they mention them in the text. E.g. in the “ *For example, in Kvik Pathways we retrieve fold change for single genes*” part in the second paragraph of the Methods section.
5. When mentioning bar charts in the Implementation section, the authors can provide a figure with a bar chart, or provide a respective link.
6. In the Use case, it is mentioned that *“the statistical significance level is indicated by the color of the node’s frame”*. However, the color code is not clear and it is also not mentioned in the Figure 1 legend, nor there is any information found in the Kvik pathways.

I have read this submission. I believe that I have an appropriate level of expertise to confirm that it is of an acceptable scientific standard, however I have significant reservations, as outlined above.

# Peer review: Referee response for version 2

**DOI:** 10.5256/f1000research.7113.r9080

**Published:** 2015-06-25

**Authors:** Paul Klemm

**Affiliations:** 1 Department Simulation and Graphics, Faculty of Computer Science, Otto-von-Guericke University Magdeburg, Magdeburg, Germany

All criticized points were considered in the revision. I think the paper has substantially improved. The distinction between `Kvik` and `Kvik Pathways` is made clear and a list of contributions makes it easier to follow the structure. I think you should consider putting the requirement analysis into a dedicated section.

I see no reason to not approve the paper in its current state.

I have noticed two minor typos:

- Abstract
  - Typo: Missing whitespace after sentence "Kvik Pathways follows the three-tier architecture in web applications using a powerful back-end for statistical analyses and retrieval of metadata."
- Implementation
  - Missing verb? "Kvik Pathways Cytoscape.js to create a pathway visualization from the list of nodes and edges and overlay the nodes on the pathway image."

Apart from the paper, I have one comment on your response on my review w.r.t. the differences regarding the NIK paper. You stated that

" *we removed the security since we believe that data should be publicly available, [...]*"

I completely agree with the notion of open data. In my experience though, epidemiologists are often bound to contracts enforcing confidentiality of the data to protect the privacy of the participants. Our project partners legally must not use a system, which does not allow for secure data handling.

This has no consequence for your paper at all, I just think that this is a observation from our collaboration with epidemiologists you might appreciate.

I have read this submission. I believe that I have an appropriate level of expertise to confirm that it is of an acceptable scientific standard.

# Peer review: Referee response for version 1

**DOI:** 10.5256/f1000research.6693.r8533

**Published:** 2015-04-30

**Authors:** Zhenjun Hu

**Affiliations:** 1 Bioinformatics Graduate Program and Department of Biomedical Engineering, Boston University, Boston, MA, USA

The manuscript presents Kvik as an open-source system  for explorative analysis of functional genomics data from large epidemiological studies. The authors seem have excellent ideas, but the implementation of the tool is far behind these ideas. I would like to approve the manuscript if the following points can be addressed:

 

1. The target of the tools. There are in general two type of tools: data provider and tool provider. The two of course can be combined. The prior in general provides knowledge, and the later provides functions to analyze users' own data. Kvik however seems lack the data to be a knowledge provider, and also does not provide enough functionality to be the later. To be the former, I will recommend authors to add more epidemiological data, to be the later, the author need to give clear instruction how user's own data can be analyzed using Kvik. For an example, the idea to connect to the cloud service is excellent, but how can Kvik to achieve this?
2. The implementation of Kvik seems to be improved, especially the performance. When I tried the Kvik, the browser tells me several time that the page is not responding. Yet I know the page is responding, but just take too much time. In addition, from data security point of view, it is not good to use RPCs from the browser layer to data engine directly, it shall be avoid in general in the three-tier architecture.
3. The manuscript need to focus more on the functionality of the tool. The current manuscript has too many technical details.

I have read this submission. I believe that I have an appropriate level of expertise to confirm that it is of an acceptable scientific standard, however I have significant reservations, as outlined above.

# Response

**Published:** 2015-06-02

**Authors:** Bjørn Fjukstad

**Affiliations:** University of Tromso, Norway

We would first like to thank the reviewer Zhenjun Hu for his thorough feedback and comments.

1. Since we have open-sourced the application we believe that Kvik Pathways can provide knowledge in itself, but also be used by other researchers to gain knowledge in their data. We have modified the use case to make it more clear how users can do so themselves. We also refer to the online repository where this information is also available. As we state in the Introduction we provide Docker images for running Kvik Pathways in a cloud service.
2. We agree, the current implementation can be improved. We are working on a second version where we have reduced the latency and that gives more feedback to the user if he has to wait. Regarding using RPCs from the browser layer we agree. We have updated the note with more details on how the system is implemented. The Data Engine provides an HTTP REST API that the browser layer queries. It does not send RPC requests from the browser layer, but from the frontend when it receives a request to the HTTP REST API. Also, the only RPCs that are allowed to run are defined in the R analysis script.
3. We agree. We have reduced the number of implementation details to make the functionality stand out more.

# Peer review: Referee response for version 1

**DOI:** 10.5256/f1000research.6693.r8165

**Published:** 2015-04-23

**Authors:** Paul Klemm

**Affiliations:** 1 Department Simulation and Graphics, Faculty of Computer Science, Otto-von-Guericke University Magdeburg, Magdeburg, Germany

The work presented by Fjukstad *et al*. pursues in pushing the notion of open science in epidemiology. It describes Kvik, a web-based tool for analyzing genomic pathways. I really like the ideas behind it and value the detailed implementation section as well as the state-of-the-art techniques used. On the other hand I think that the paper should focus more on describing the epidemiological context, associated requirements and target groups to communicate the design choices for Kvik. Go into detail on the application *workflow.*

**General Feedback**

I like the approach the authors take with the paper. The tool they describe seems to be well suited for analyzing genomic pathways in the epidemiological context.

I miss a clear statement on the papers contribution. Maybe you can put a bullet list in the *Introduction* section to tell the reader what things can be done with your software, which could not be done before!

In my understanding, epidemiology is a very interdisciplinary in terms of associated experts. You have your clinicians deriving hypotheses from their day to day practice, statisticians deriving statistically sound conclusion as well as biologists and computer scientists associated with such projects. *Which of these are your target group?*

When you described your target users, describe what they are trying to find out. How does your tool help them doing that? Does it allow them do their work faster? Do they derive insights they could not get before? The latter would be a huge contribution! Please give more details on the *workflow* of you system!

I miss a clear distinction from the NIK-2014 paper " Kvik: Interactive exploration of genomic data from the NOWAC postgenome biobank". The paper was also not cited in this work. It seems to me that the majority of the content of the presented paper can already be found in the NIK-2014 paper. Please elaborate on the differences and cite the paper. *If you can not state clear differences, there is, in my opinion, no point in publishing this paper and I will rate it 'Not Approved'*.

**Title**

The title of the work is appropriate.

**Abstract**

The abstract motivates the need for new tools, which allow to assess the vast amount of epidemiological data well. In my opinion it can be improved by:

- reduce the amount of implementation detail. You tell the reader later on which frameworks and libraries you use
- explain who are your users.
- what can be done with your tool now, which could not be done before?

Minor comments on the abstract:

- "Existing data exploration tools do not provide all the required functionality for such multi-study data exploration." This is a dangerous statement, since you do not say anything about what the *required functionality* is! I think I know what you are trying to get at, in the introduction you describe it better with: "Although an existing tool may be useful for one project, no tool provides the required functionality for several different projects."

**​Introduction​**

The introduction can be improved by clearly stating the contributions (e.g., as bullet points).

I would like to see some reference or a method on how the five requirements were acquired. These are all things, which are important in almost all applications. Where is the difference of software in the epidemiological context towards other context and how does Kvik adapt to the arising requirements? You answer many of these questions, later on when you repeat all the requirements again, but to me it is not structured well.

**Methods**

The method section is written well. I would like to know how the users modify the R scripts (beginning second paragraph). Do they do this inside Kvik or do they have to switch into another software for it?

Figure 1 caption: What can the user do now after he or she selected the gene? The workflow is not clear to me.

Figure 2 was already presented very similarly in the NIK'14 paper.

Minor: Closing parenthesis in sentence "In our setup the Data Engine in the back-end layer provides an interface to the NOWAC data repository stored on a secure server on our local Stallo Supercomputer Table 1 provides the interfaces)."

**Use Case**

The use case section can be strengthened by reducing the amount of implementation details (in my opinion mentioning the individual function names is not necessary to comprehend the functionality) and focusing more on the involved actors and tasks and contexts associated with the use case. What feedback was given by the user(s)?

**Reusability**

The effort of the authors to make the software publicly available is worth a special note. Modern state of the art techniques are combined with powerful back-end systems, which scale well on different application scenarios.

I have read this submission. I believe that I have an appropriate level of expertise to confirm that it is of an acceptable scientific standard, however I have significant reservations, as outlined above.

# Response

**Published:** 2015-06-02

**Authors:** Bjørn Fjukstad

**Affiliations:** University of Tromso, Norway

We would first like to thank the reviewer Paul Klemm for his thorough feedback and comments.

**Difference between the NIK paper and the Application Note**

When we wrote the NIK paper, Kvik was only a system for exploring genomic data in the context of pathways. Since then we have realized that exploring genomic data in pathways is not enough, that we need a framework that allows us to build different applications for exploring data from different studies with different designs. With this in mind we have refined the requirement analysis from our initial Kvik system and developed more general requirements that these applications should satisfy.

From our initial Kvik implementation we have now decoupled the application (Kvik Pathways) from the framework, allowing fast development of new applications. The Kvik framework provides interfaces to a Data Engine that provides statistical analyses, and interfaces to online databases such as KEGG. Kvik Pathways is the first application that we developed using the Kvik Framework. Using Kvik we have developed several other applications that will be published in the near future.

In the NIK paper we described from a computer science point of view the features of Kvik, both looking at the application itself, and the backend features that are now a apart of the Kvik Framework. The NIK paper was written to give a more in detail view of how the system works and performs, while in this application note we want to describe how our epidemiology researchers helped to develop the application and how they used it to reproduce results they found in an already published dataset.

Using an already published dataset was important to us since it allows us to provide a publicly available Kvik installation for others to use. We will revisit the second paragraph of the Use case section where we discussed how we used the initial Kvik system to explore different data from a different study design.

To sum up, our new contributions in the application note are as follows:

- Publicly available application
- Publicly available Docker containers that researchers can use to set up local installations of Kvik Pathways.
- Reproduced the results from an already published dataset to make the system publicly accessible at kvik.cs.uit.no
- A more refined requirement analysis that reflects our experiences after publishing the NIK paper. The important changes are:
  i) emphasis on integration of online knowledge bases (interactive requirement),
  ii) emphasis on the system being flexible to adapt to data and different statistical analyses,
  iii) we removed the security since we believe that data should be publicly available, and
  iv) put emphasis on separating computation and visualization (lightweight).

We have cited the NIK paper in the application note and improved the text to highlight the differences between the framework and the Kvik Pathways application.

We have included a list of contributions in the Introduction section.

We agree that epidemiology is a very interdisciplinary. Kvik Pathways has been developed in a team of epidemiologists, biologists, statisticians and computer scientists. The application note targets such groups of researchers working together to develop systems for gaining biological insights in genomic data. We have re-written parts of the note to clarify how researchers have used the application.

**Abstract : **We agree and have reduced the amount of implementation detail and made it more specific what our users have done with the application.

Introduction: We have revisited the requirements and specified how these are different from regular applications. We believe that it is best to separate the requirements and how we solved them in two different lists.

**Methods: **As of today users modify R scripts outside Kvik. We have made it clear in the methods section. We will expand Figure 1 caption to clearify the workflow. Regarding figure 2. We chose to include it since it highlights the important three-tiered architecture with applications that use the Kvik framework. We have modified the figure to highlight connection between the application and the framework.

**Use case: **As mentioned we will expand this section with a more detailed workflow. Since it is an app note targeted towards users we will reduce implementation details and refer to the source code.
